# Supplementary material for: Adoptive cancer immunotherapy using DNA-demethylated T helper cells as antigen-presenting cells
Source: Nat Commun. 2018 Mar 6;9:785. doi: 10.1038/s41467-018-03217-9 (PMC5840134; doi:10.1038/s41467-018-03217-9)
Supplement: Supplementary file 1 — Supplementary Information [file 41467_2018_3217_MOESM1_ESM.pdf]

Supplementary Information

Kirkin *et al*. Adoptive cancer immunotherapy using DNA-demethylated T helper cells as antigen-presenting cells

Table of Contents

Section	Page
Supplementary Methods	4
MRI	4
O-(2-[^{18}F]fluoroethyl)-L-tyrosine positron emission tomography (FET-PET) imaging	5
Radiolabeling and scintigraphy of leukocytes (control) and cytotoxic lymphocytes	6
Safety and tolerability	7
Supplementary Figure 1. Low induction of the stress proteins in DC-activated lymphocytes after 5-aza-CdR-treatment	8
Supplementary Figure 2. Derepression of CT antigens in DC-induced lymphocytes by 5-aza-CdR	9
Supplementary Figure 3. Derepression of CT antigens measured by quantitative PCR (qPCR)	10
Supplementary Figure 4. Duration of CT-antigen expression after induction by 5-aza-CdR	11
Supplementary Figure 5. Derepression of CT antigens by 5-aza-CdR in $CD4^+$ and $CD8^+$ T cells.	12
Supplementary Figure 6. Specificity of the 57B monoclonal antibody (sandwich ELISA)	13
Supplementary Figure 7. Immunoblot analysis of MAGE expression in 5-aza-CdR-treated DC-induced lymphocytes	14
Supplementary Figure 8. Demethylation of CT-antigen promoters in dendritic cell-induced lymphocytes	15
Supplementary Figure 9. Growth of PBLs and 5-aza-Cdr-treated TH cells cultured separately and in co-culture	16
Supplementary Figure 10. Recognition 5-aza-Cdr-treated CD4+ TH cells by cytotoxic lymphocytes	17
Supplementary Figure 11. Effect of a blocking antibody to HLA class I (W6/32) on CTL recognition of 5-aza-CdR-treated TH cells	18
Supplementary Figure 12. CTL-mediated recognition of breast cancer cells and immortalized melanocytes (Mel-ST)	19
Supplementary Figure 13. Effect of a blocking antibody to HLA class I (W6/32) on CTL recognition of MDA-MB-231 breast cancer cells	20
Supplementary Figure 14. CTL-mediated lysis of MDA-MB-231 breast cancer cells	21
Supplementary Figure 15. Real-time monitoring of CTL-mediated cytotoxicity	22

Supplementary Figure 16. Tumor-specific cell lysis of breast cancer cells	23
Supplementary Figure 17. Immunohistochemical analysis of MAGE-A3 in glioblastoma biopsies at diagnosis	24
Supplementary Figure 18. Flow diagram summarizing the trial flow and grounds for exclusion from the study	25
Supplementary Figure 19. Transient changes in circulating lymphocyte, leukocyte and neutrophil counts after injection of cytotoxic lymphocytes	26
Supplementary Figure 20. Tumor response in Patient 20	28
Supplementary Figure 21. T cells at the previous tumor site in Patient 6 (autopsy material)	29
Supplementary Table 1. Proportion of $CD4^+$ cells in non-adherent lymphocyte cultures after induction with mature DCs or PHA	30
Supplementary Table 2. Expression of selected surface markers on NK and T cells in the final product	31
Supplementary Table 3. Stimulatory activity of $CD4^+$ and $CD8^+$ cells after treatment with 5-aza-Cdr	32
Supplementary Table 4. Lysis of MEL-ST cells expressing single CT antigens by CD56-depleted cytotoxic lymphocytes	33
Supplementary Table 5. Baseline characteristics of glioblastoma patients (N = 25)	34
Supplementary Table 6. Expression of CTCFL and MAGE-A3 in diagnostic tumor biopsies (immunohistochemical analysis)	35
Supplementary Table 7. Induction of MAGE-antigen mRNA expression by 5-aza-CdR in TH cells from patients with glioblastoma	36
Supplementary Table 8. Cell therapy products administered in the phase 1 glioblastoma trial	38
Supplementary Table 9. Adverse events reported during the 20-week study period after first injection of therapeutic cells	39
Supplementary Table 10. Serious adverse events reported during the 20-week study period after first injection of therapeutic cells	40
Supplementary Table 11. Additional injections of cytotoxic lymphocytes under approved compassionate use	41
Supplementary Table 12. CT-antigen expression in cancer cell lines used in this study	42
Supplementary Table 13. Antibodies	43
Supplementary Table 14. Primer sequences for RT-PCR	44
Supplementary Table 15. Primer sequences for bisulfite pyrosequencing	44
Supplementary Table 16. Primer sequences for MS-MCA	44
Supplementary References	45

Supplementary Methods

MRI. The images were acquired on a 1.5 tesla MR unit (Siemens Magnetom Avanto syngo MR B17, Siemens Medical Systems, Erlangen, Germany). The MR imaging included the following sequences: 1) Pre-contrast MPR, sagittal T1 (TR/TE = 1930/2.92 ms), FoV 256 mm, slice thickness 1.00 mm, 2) Axial diffusion (TR/TE= 3400/102 ms), slice thickness 5.0 mm, average ADC maps, 3) Coronal TIRM, dark-fluid (TR/TE = 9000/77.0 ms), slice thickness 5.0 mm, 4) Axial T2 (TR/TE = 4000/107 ms), slice thickness 5.0 mm and post-contrast MPR as sequence 1). The contrast used was MultiHance® (Bracco S.p.A, Milano, Italy). A dose of 0.2 mL/kg body weight (maximum dose 15 mL) was administered intravenously as a bolus after acquisition of sequence 1-3. Qualitative assessment was performed for post-contrast T1 sequences only.

O-(2-[^{18}F]fluoroethyl)-L-tyrosine positron emission tomography (FET-PET) imaging. A single frame static PET acquisition was performed 20 to 40 min p.i. of 200 MBq FET on a Biograph 64 Truepoint PET/CT-scanner (Siemens, Knoxville, USA). Images were attenuation and scatter corrected, and reconstructed with OSEM 3D (6 it, 16 sub, 5 mm Gauss). All patients fasted for at least 6 h before FET injection. FET PET images were co-registered to the post-contrast T1- and FLAIR/T2-weighted MRI. A background region of interest (ROI) was drawn in a healthy appearing cortical region in the unaffected hemisphere contralateral to the tumor. The tumor was auto-contoured in 3D to include voxels ≥ 1.6 x background according to established criteria[1] enabling the assay of tumor activity by the metabolically active tumor volume (FETvol), and the ratio between maximal tumor activity and background (Tmax/background).

Radiolabeling and scintigraphy of leukocytes (control) and cytotoxic lymphocytes. Leukocytes were labeled with ^{111}In-tropolone. In brief, 85 ml of venous blood was drawn and anticoagulated with citric acid–glucose. Methylcellulose (2% (w/v)) was added to the blood for faster sedimentation of erythrocytes. Cell-rich plasma was transferred to another tube and leukocytes were isolated by gradient centrifugation. Isolated leukocytes were resuspended in 1 ml of cell-free plasma and added to a mixture of 20–30 MBq of $^{111}InCl_3$ and 0.1 ml of 0.0044 M tropolone. Following centrifugation, resuspension and quality control (labeling efficiency), a dose of 9–12 MBq of labeled leukocytes was injected intravenously within 1 h after labeling. Cytotoxic lymphocytes were labeled with ^{111}In-tropolone and handled in a similar way as leukocytes, except that they were suspended in 1-2 ml of Plasma-Lyte (Baxter International, Deerfield, IL). In two patients, cytotoxic lymphocytes were labeled with ^{99m}Tc labeled hexamethylpropyleneamine oxime (HMPAO) using the same steps as described above except that the mixture added to the cells was 900-1100 MBq of ^{99m}Tc-HMPAO (Ceretec, Garden Grove, CA, USA), and a dose of 150-200 MBq of labeled cells was injected intravenously. Whole body scintigraphy was obtained 2-4 h and 20–24 h and single-photon emission computed tomography / computed tomography (SPECT/CT) of the head was obtained 24 h after injection of the labeled cells using a SPECT/CT camera (Precedence, Philips, The Netherlands). SPECT images were attenuation and scatter corrected, and reconstructed with Astonish (4 it, 16 sub, Hanning). The scans were classified as negative, when no sites of abnormal uptake were observed, or as positive when at least 1 focus of abnormal uptake was observed. Uptake of an intracranial focus seen on SPECT/CT was compared to background in the contralateral area (tumor/background ratio). All positive foci were correlated to MRI and FET-PET scans to resolve whether a focal uptake was related to tumor (true positive) or unspecific uptake to non-tumor tissue (false positive).

Safety and tolerability. Blood pressure, pulse, and body temperature were measured 15 min, and 1, 4, 8, 12, 18, 24, 30, 36, 42 and 48 h after the first treatment and until 24 h after the second and third treatments. Karnofsky Performance Score was measured at weeks 4, 6, 9, 14 and 20. Blood samples were obtained at each admission and analyzed for leukocytes (differential count), C-reactive protein (CRP), hemoglobin, electrolytes, renal function, liver count (liver enzymes). Adverse events observed by the investigator, or spontaneously reported by the patient, were registered during the entire study period.

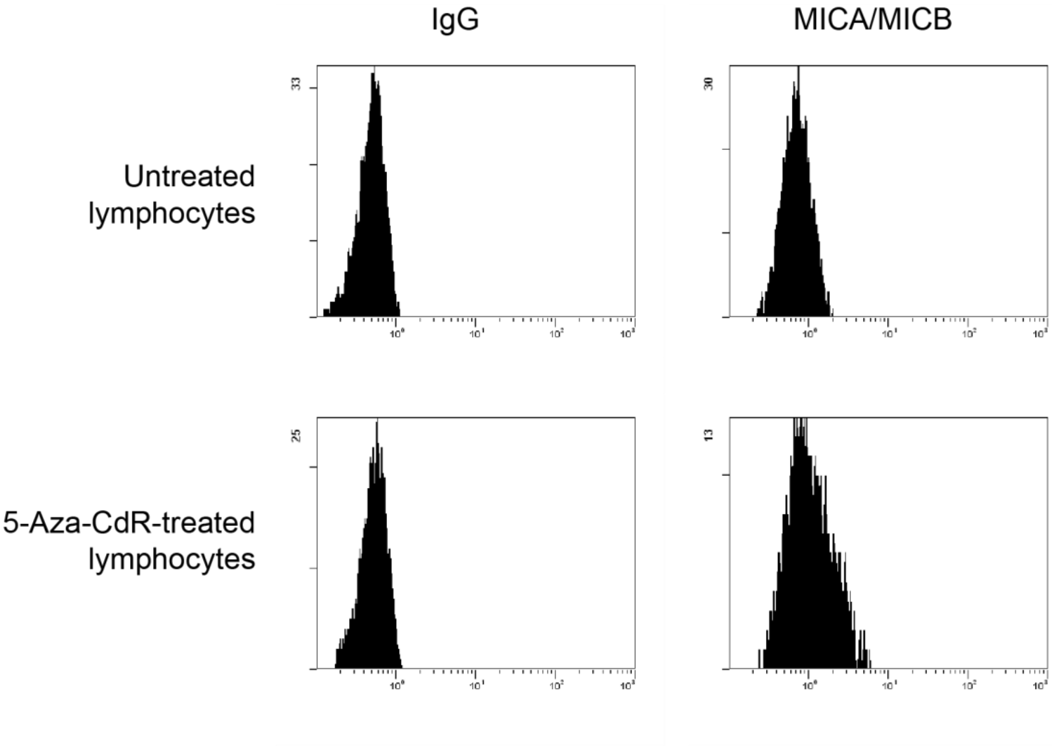

Supplementary Figure 1. Low induction of the stress proteins in DC-activated lymphocytes after 5-aza-CdR treatment.
A representative lymphocyte culture was grown for 6 days in the presence of fully mature DCs and treated by 10 µM 5-aza-CdR for 2 days. Untreated lymphocytes were analyzed as control. Expression of MICA and MICB on these cells was assessed by flow cytometry.

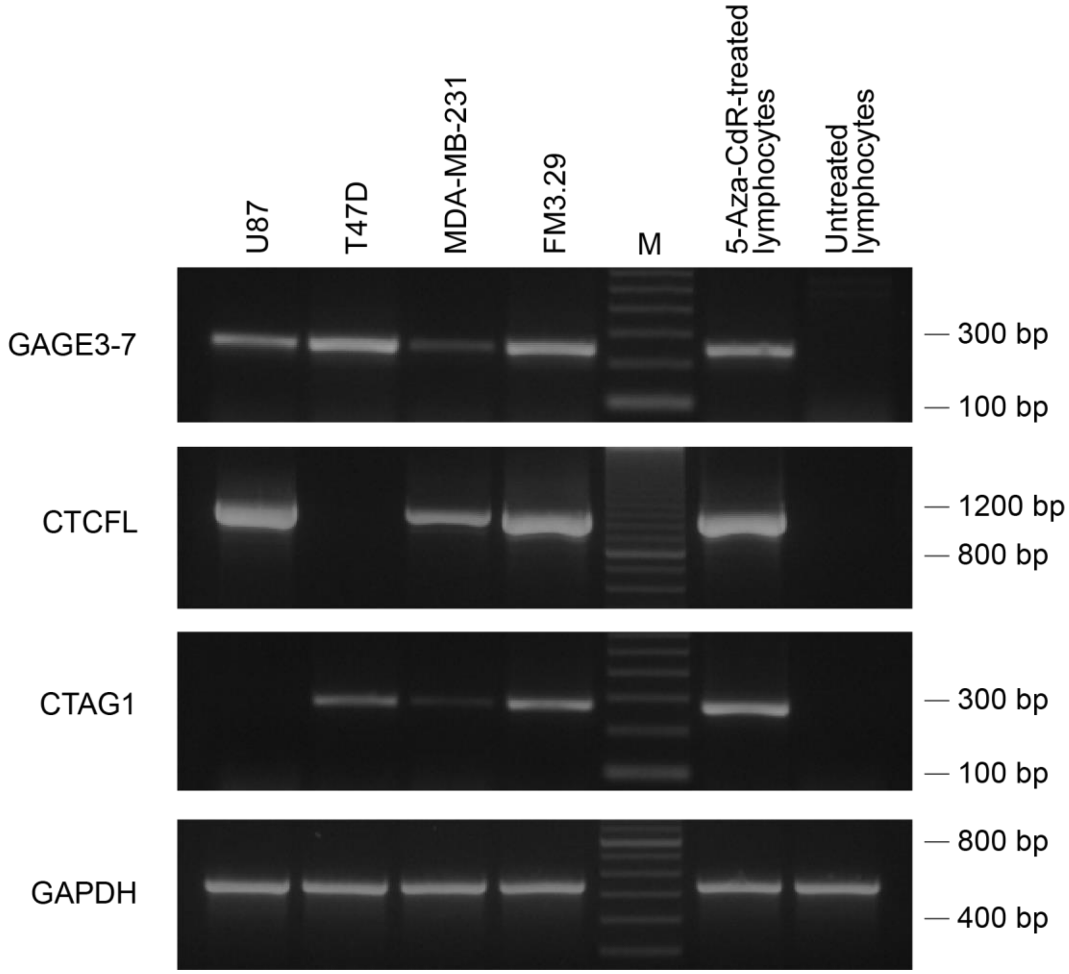

Supplementary Figure 2. Derepression of CT antigens in DC-induced lymphocytes by 5-aza-CdR.
DC-activated lymphocytes were treated with 10 µM 5-aza-CdR for 2 days. Expression of GAGE, CTCFL (also known as BORIS) and CTAG1 (also known as NY-ESO-1) mRNA was analyzed by RT-PCR. GAPDH expression was analyzed as a control. T47D (breast cancer), MDA-MB-231 (breast cancer) and FM3.29 (melanoma) cells were included as examples of tumor cells expressing these CT antigens. M, 100-bp ladder.

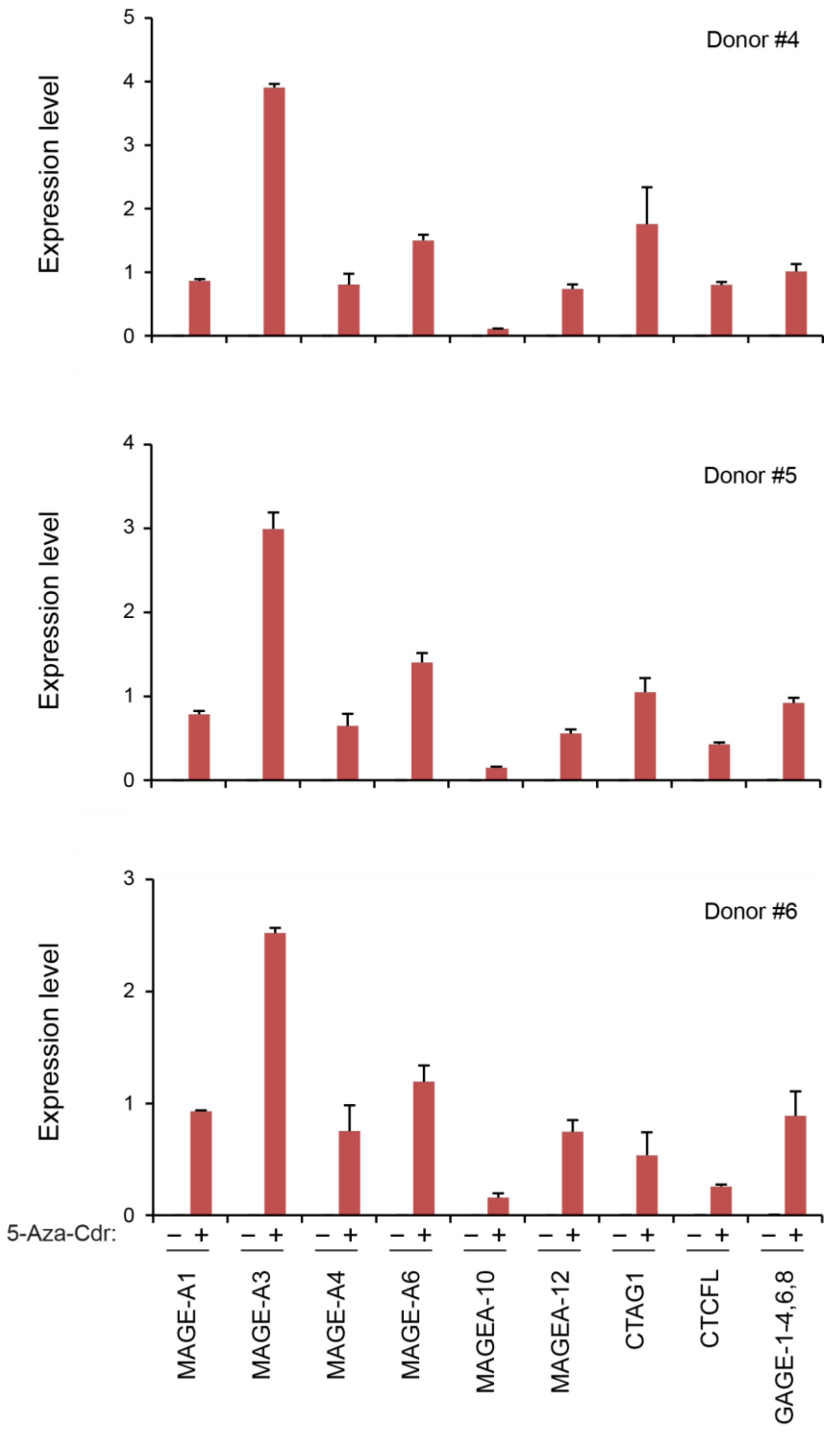

Supplementary Figure 3. Derepression of CT antigens measured by quantitative PCR (qPCR).
DC-activated lymphocyte cultures from three healthy donors were grown in the presence (+) or absence (-) of 10 µM 5-aza-CdR for 2 days. Expression levels of CT-antigen mRNA were determined relative to GAPDH. Data of triplicates are presented as mean ± s.d.

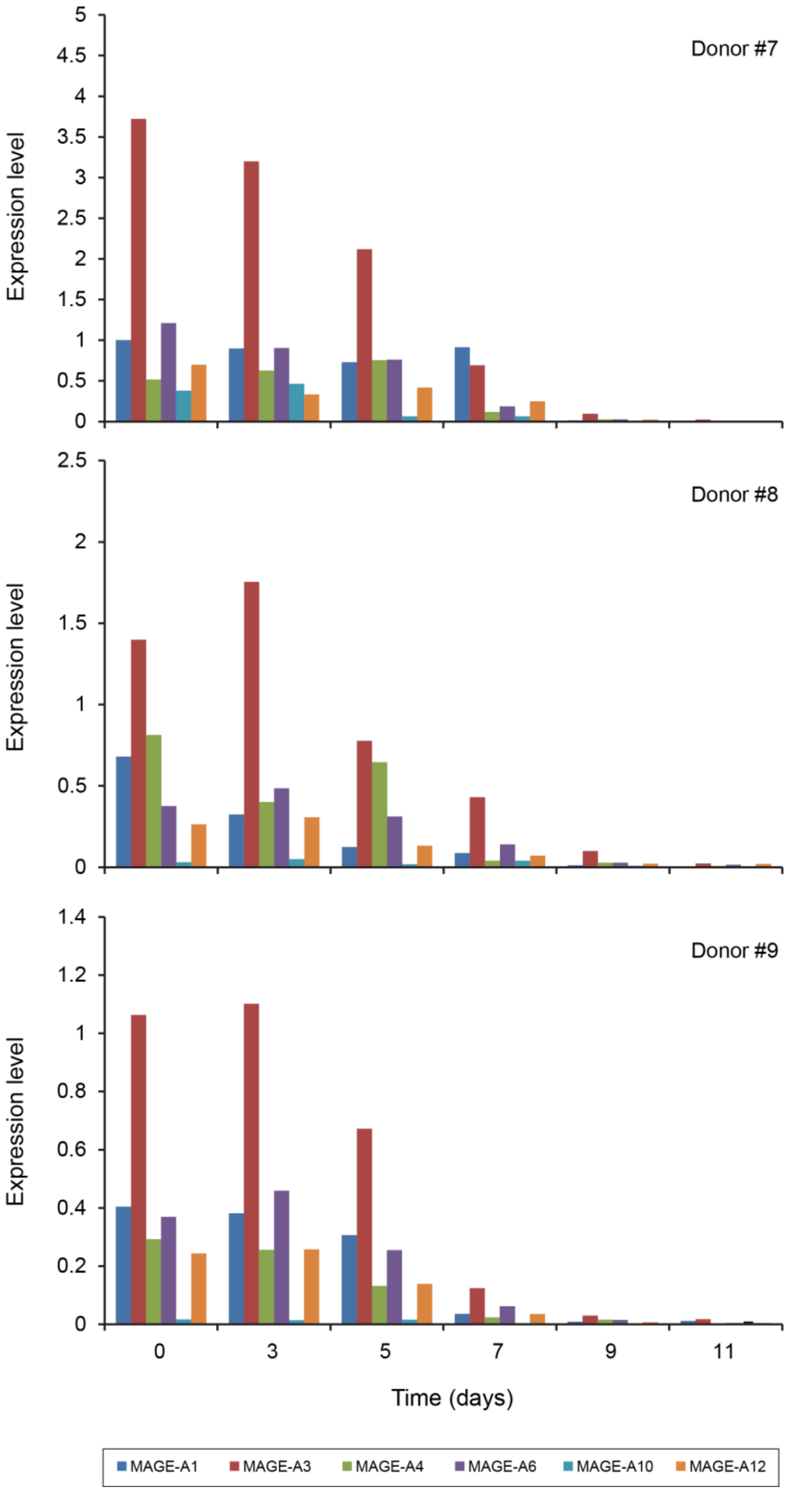

Supplementary Figure 4. Duration of CT-antigen expression after induction by 5-aza-CdR.
DC-activated lymphocyte cultures from three healthy donors were grown in the presence of 10 µM 5-aza-CdR for 2 days. Post-5-aza-CdR treatment expression levels of CT-antigen mRNA were determined relative to GAPDH using qPCR.

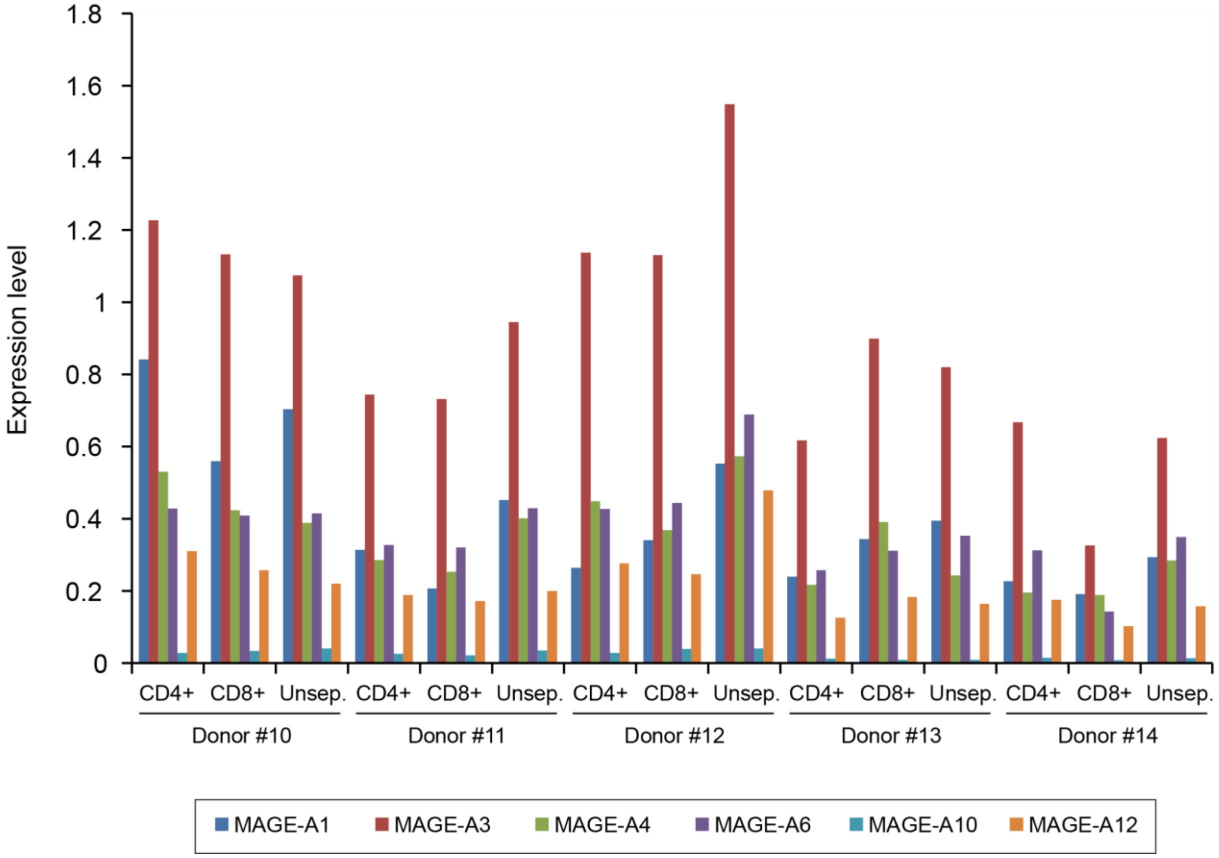

Supplementary Figure 5. Derepression of CT antigens by 5-aza-CdR in CD4^{+} and CD8^{+} T cells.
DC-activated lymphocyte cultures from three healthy donors were grown in the presence of 10 µM 5-aza-CdR for 2 days. CD4^{+} and CD8^{+} cells were isolated by positive selection using human CD4 and CD8 microbeads (Miltenyi Biotec), respectively. Expression levels of CT-antigen mRNA were determined relative to GAPDH using qPCR.

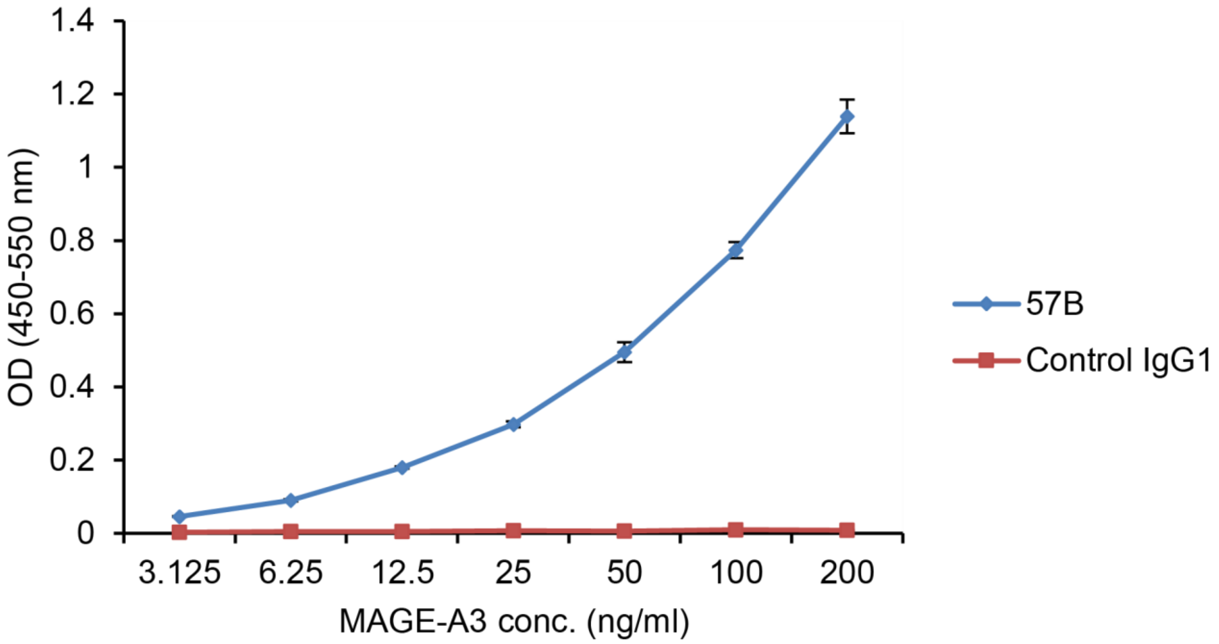

Supplementary Figure 6. Specificity of the 57B monoclonal antibody (sandwich ELISA). 96-well Maxisorb plates were covered with affinity-purified rabbit anti-MAGE-A3 antibody (0.6 µg/ml; Merck) and incubated overnight at 4°C. The plate was blocked by Assay Diluent (AD, e-Bioscience) for 2 h at room temperature and washed with phosphate buffer saline with 0.05% Tween 20 (PBS-T). Recombinant MAGE-A3 (Abcam) was applied to the wells, and after incubation for 2 h at room temperature, the wells were probed with mouse monoclonal 57B antibody or an equal amount of isotype-matched control IgG_{1k}. After incubation with 1000-fold diluted horseradish peroxidase (HRP)-labelled rabbit anti-mouse immunoglobulins (Dako), the enzymatic colorimetric reaction with TMB substrate was performed. The reaction was terminated with 3 N sulfuric acid, and the soluble product was measured using an ELISA reader as the difference between optical density at 450 nm and 550 nm. Data represent the mean ± s.d. of triplicate samples and are representative of two independent experiments.

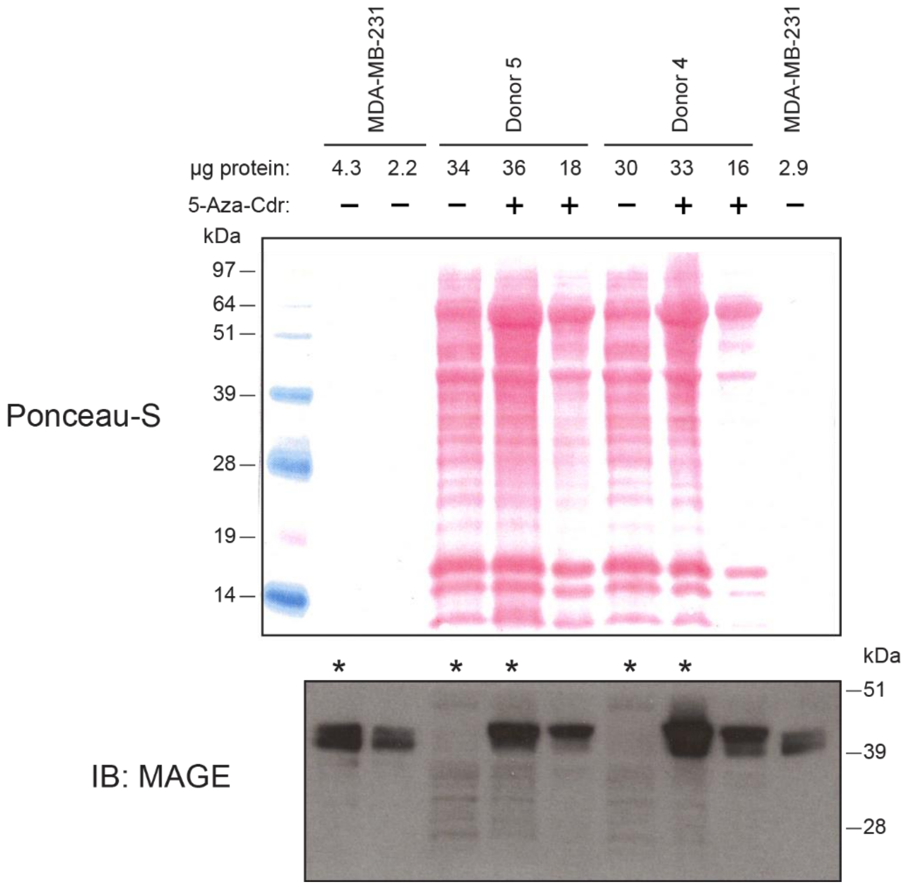

Supplementary Figure 7. Immunoblot analysis of MAGE expression in 5-aza-CdR-treated DC-induced lymphocytes. SDS lysates of MDA-MB-231 cells and untreated and 5-aza-CdR-treated DC-induced lymphocytes from two healthy donors were separated in a 12% Bis-Tris polyacrylamide gel, blotted and probed with the mouse monoclonal anti-MAGE antibody 57B. Note that the amount of protein analyzed was approximately 10 times higher for lymphocytes than for MDA-MB-231 cells (total protein per well is indicated above the Ponceau-S-stained blot). Lanes indicated by an asterisk are shown in Fig. 2D.

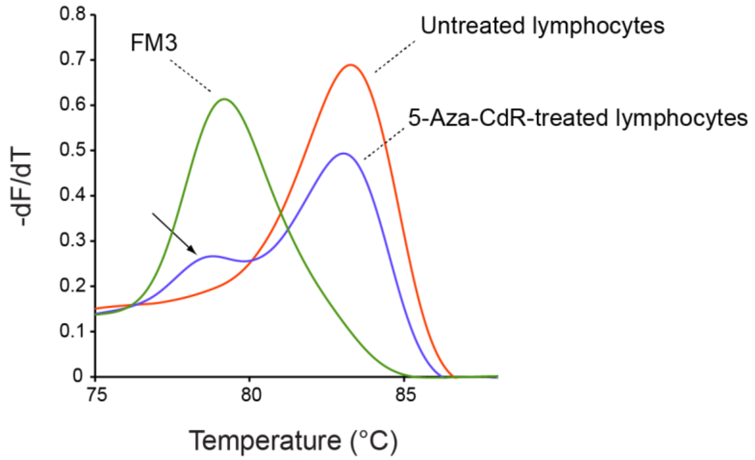

Supplementary Figure 8. Demethylation of CT-antigen promoters in dendritic cell-induced lymphocytes. Shown is the profiling of *MAGEA3/6* by bisulfite melting curve analysis in lymphocytes treated with 10 µM 5-aza-CdR for 3 days. The arrow indicates hypomethylated *MAGEA3/6* alleles in 5-aza-CdR-treated lymphocytes. FM3 melanoma cells were included as a control for hypomethylation.

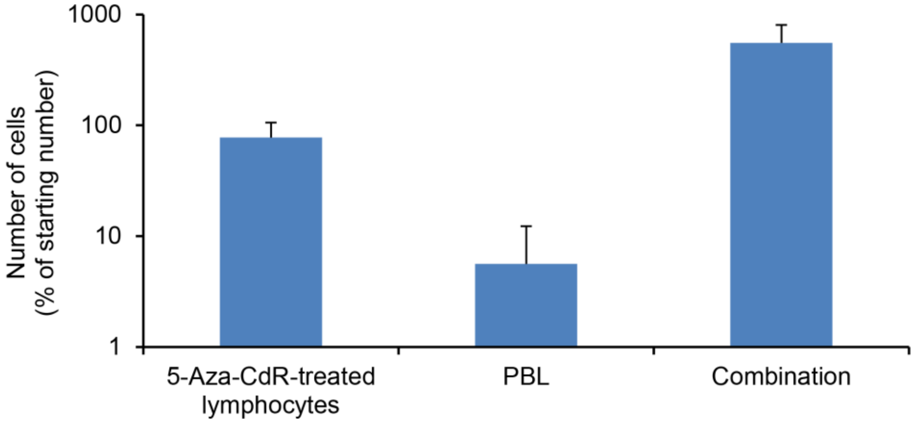

Supplementary Figure 9. Growth of PBLs and 5-aza-Cdr-treated T_H cells cultured separately and in co-culture. All cultures were incubated for 11 days under the same conditions with the addition of 25 IU/ml IL-2. The number of cells in the initial cultures was set at 100%. Data represent the mean ± s.d. of four independent experiments.

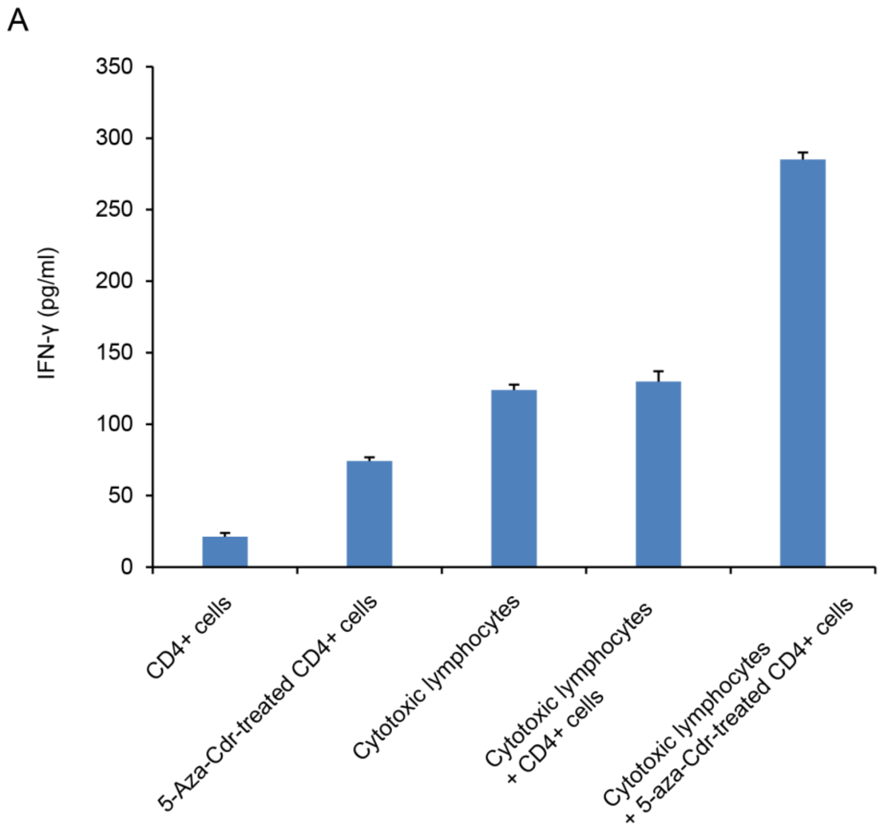

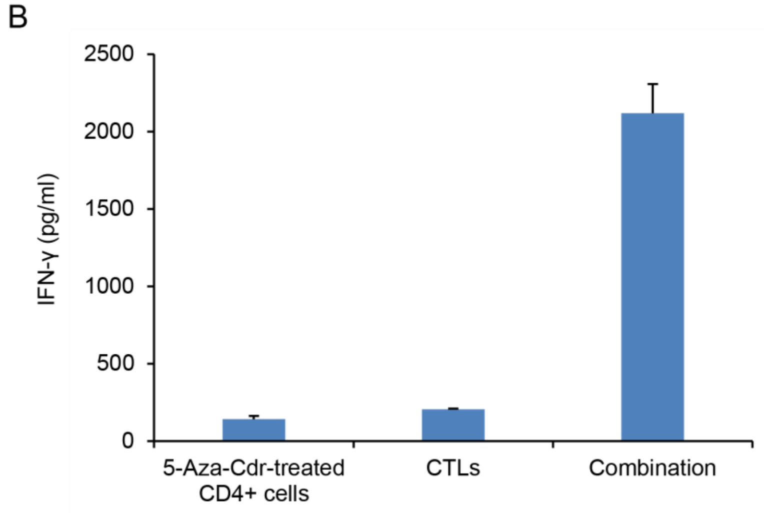

Supplementary Figure 10. Recognition 5-aza-Cdr-treated CD4$^+$ T_H cells by cytotoxic lymphocytes. Cytotoxic lymphocytes generated by exposure to 5-aza-CdR-treated T_H cells were incubated with 5-aza-Cdr-treated or untreated CD4$^+$ T_H cells for 18-20 hours. (A) Unseparated cytotoxic lymphocytes. (B) CD56-depleted lymphocytes (CTLs). A and B represent independent experiments. Data of triplicates are represented as mean ± s.d.

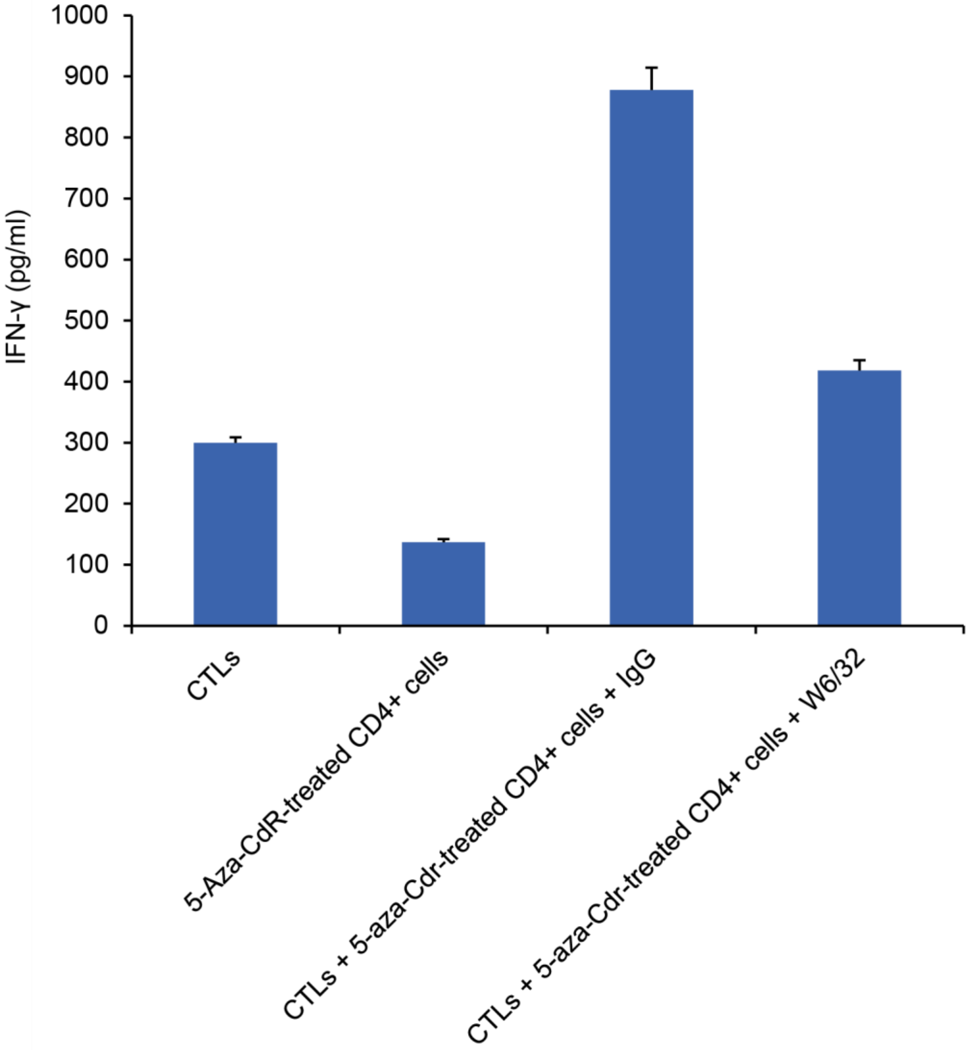

Supplementary Figure 11. Effect of a blocking antibody to HLA class I (W6/32) on CTL recognition of 5-aza-CdR-treated T_H cells. CD56-depleted cytotoxic lymphocytes were incubated with 5-aza-CdR-treated T_H cells in the presence of 10 µg/mL W6/32 or 10 µg/mL control IgG for 20 h. Data represent the mean ± s.d. of triplicate samples and are representative of three independent experiments.

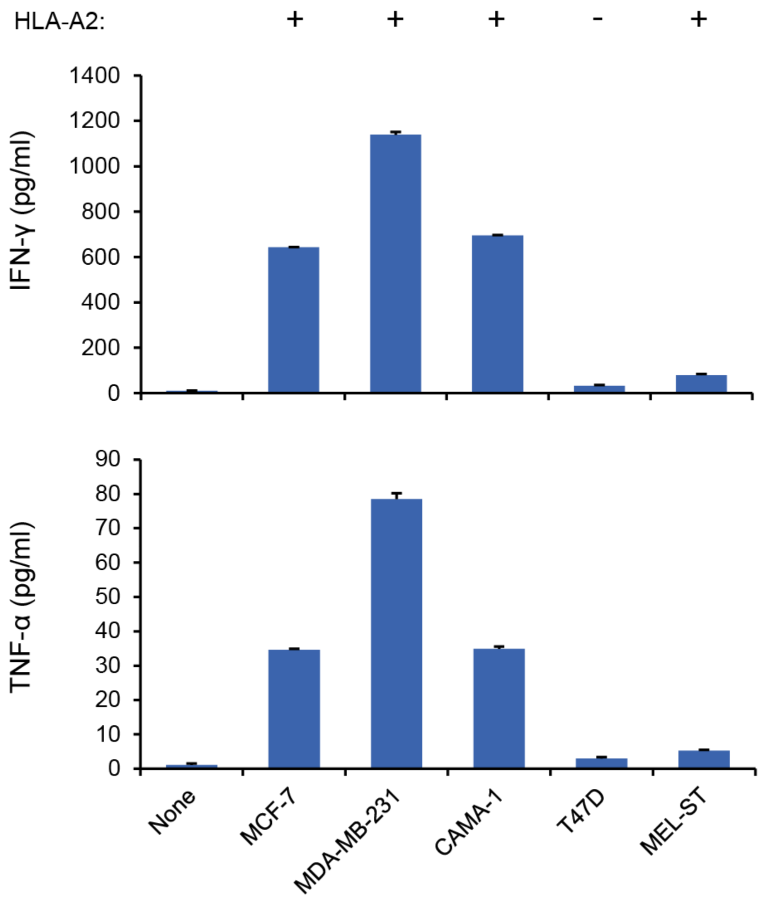

Supplementary Figure 12. CTL-mediated recognition of breast cancer cells and immortalized melanocytes (Mel-ST). Shown are the levels of IFN-γ and TNF-α production by CD56-depleted cells from a healthy HLA-A2^{+} donor during co-cultivation for 18-20 hours with the indicated cell lines. Data represent the mean ± s.d. of triplicate samples and are representative of three independent experiments.

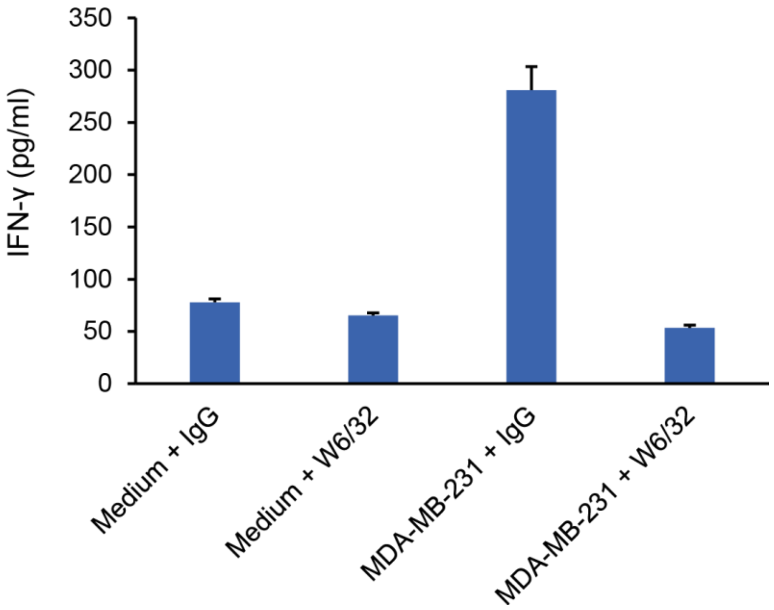

Supplementary Figure 13. Effect of a blocking antibody to HLA class I (W6/32) on CTL recognition of MDA-MB-231 breast cancer cells. CD56-depleted cytotoxic lymphocytes were incubated with MDA-MB-231 cells in the presence of 10 µg/mL W6/32 or 10 µg/mL control IgG for 20 h. Data represent the mean ± s.d. of triplicate samples and are representative of two independent experiments.

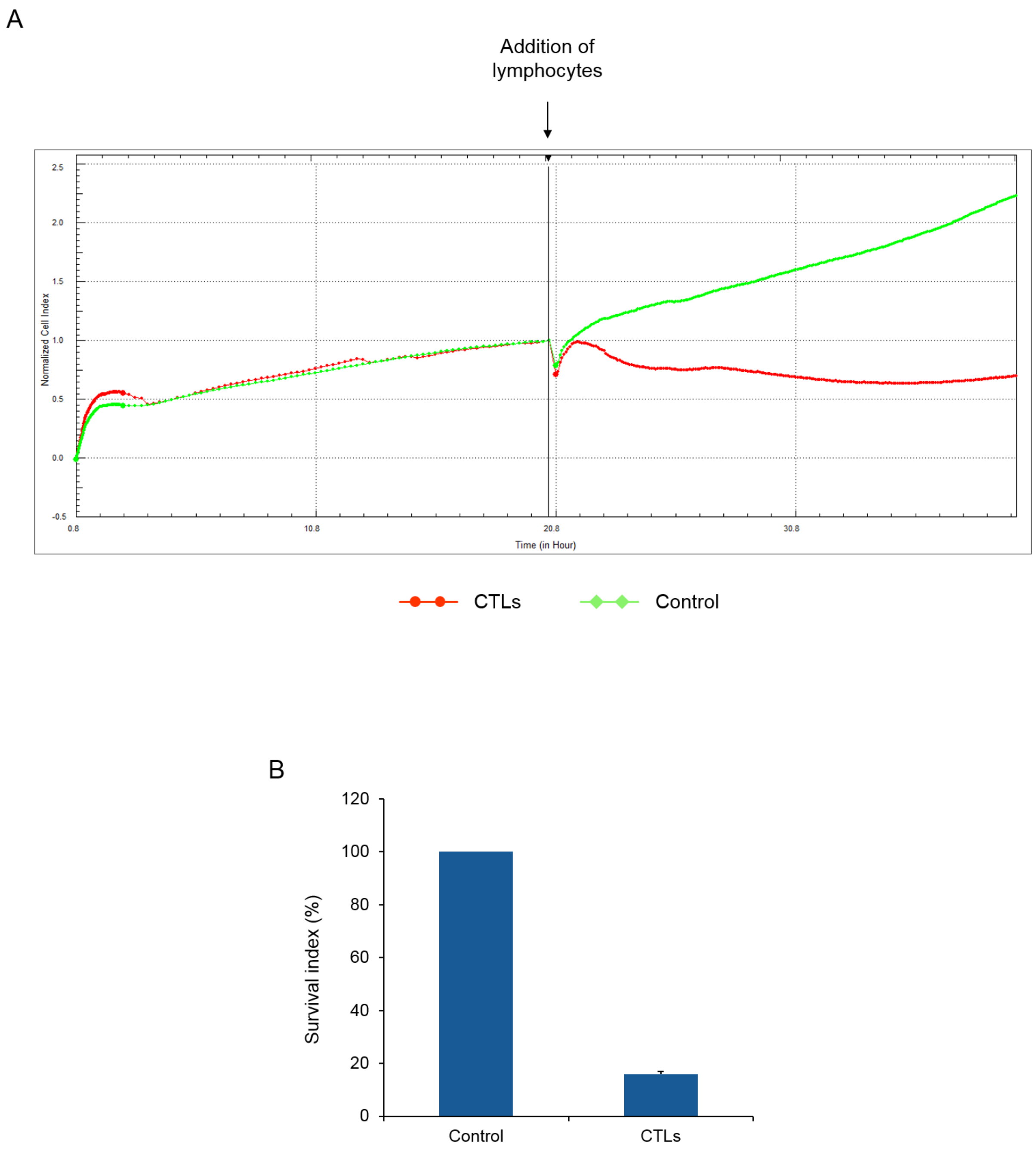

Supplementary Figure 14. CTL-mediated lysis of MDA-MB-231 breast cancer cells. (A) Real-time cell analysis using the using the iCELLigence system. MDA-MB-231 cells were seeded in L8 plates and cultured for 20 h. CTLs or medium (control) were then added, and the Cell Index was monitored for the next 20 h. (B) The same experiment showing the fraction of remaining MDA-MB-231 cells, as determined using the SRB assay.

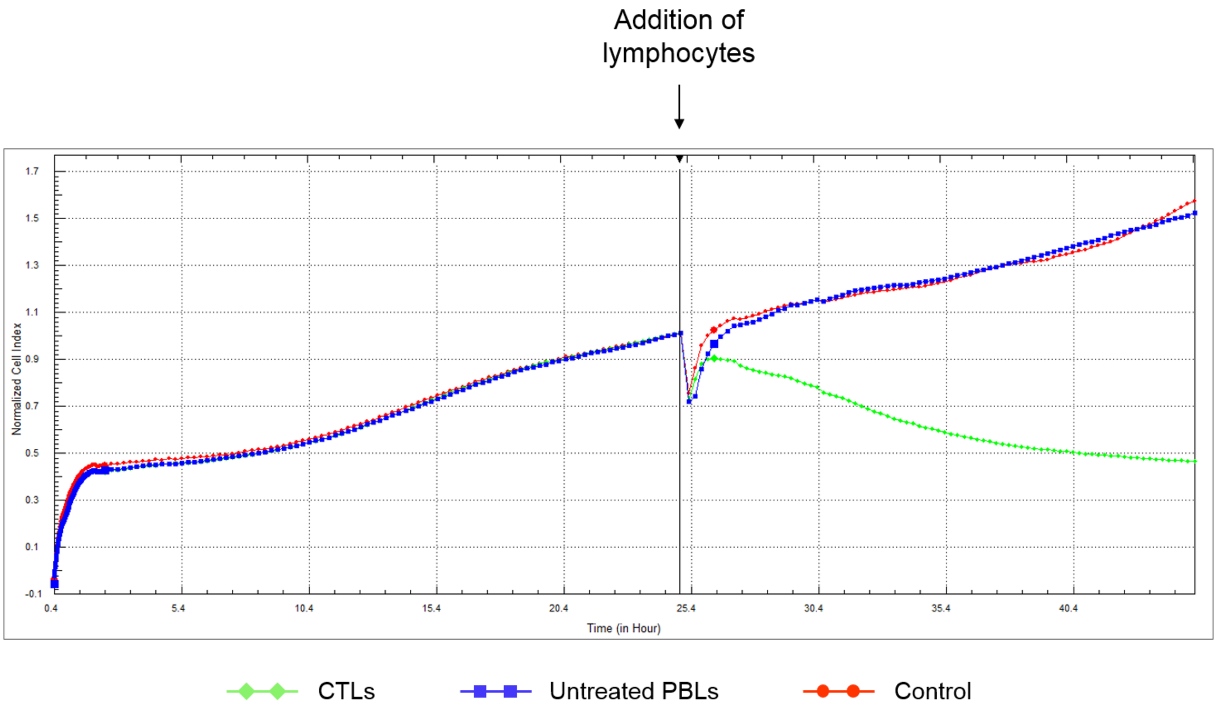

Supplementary Figure 15. Real-time monitoring of CTL-mediated cytotoxicity. MDA-MB-231 cells were seeded into L8 plates and cultured for 25 h. CTLs, PBLs cultured in the absence of 5-aza-Cdr-treated T_H cells, or medium (control) were added, and the Cell Index was monitored for the next 20 h, using the iCELLigence system.

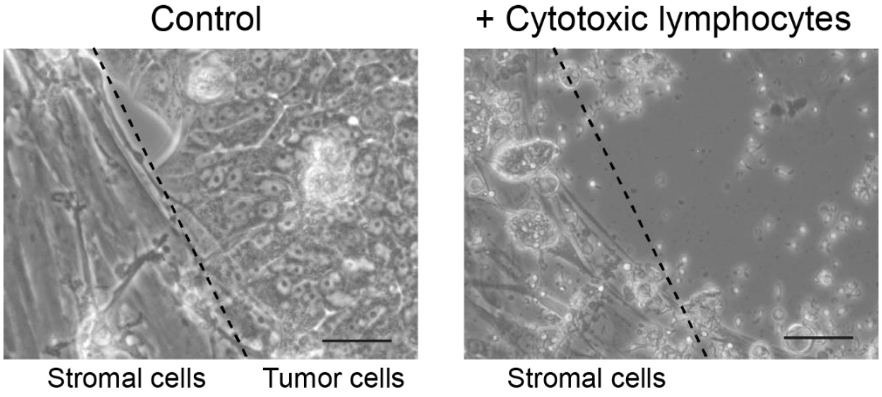

Supplementary Figure 16. Tumor-specific cell lysis of breast cancer cells. A cell culture containing tumor cells and stromal cells was established from biopsy material from a patient with breast cancer, × 40 objective; scale bar, 50 µm. Tumor-specific cell lysis was observed after incubation with autologous cytotoxic lymphocytes (CD8+ and NK cells) for 3 days, × 20 objective; scale bar, 100 µm.

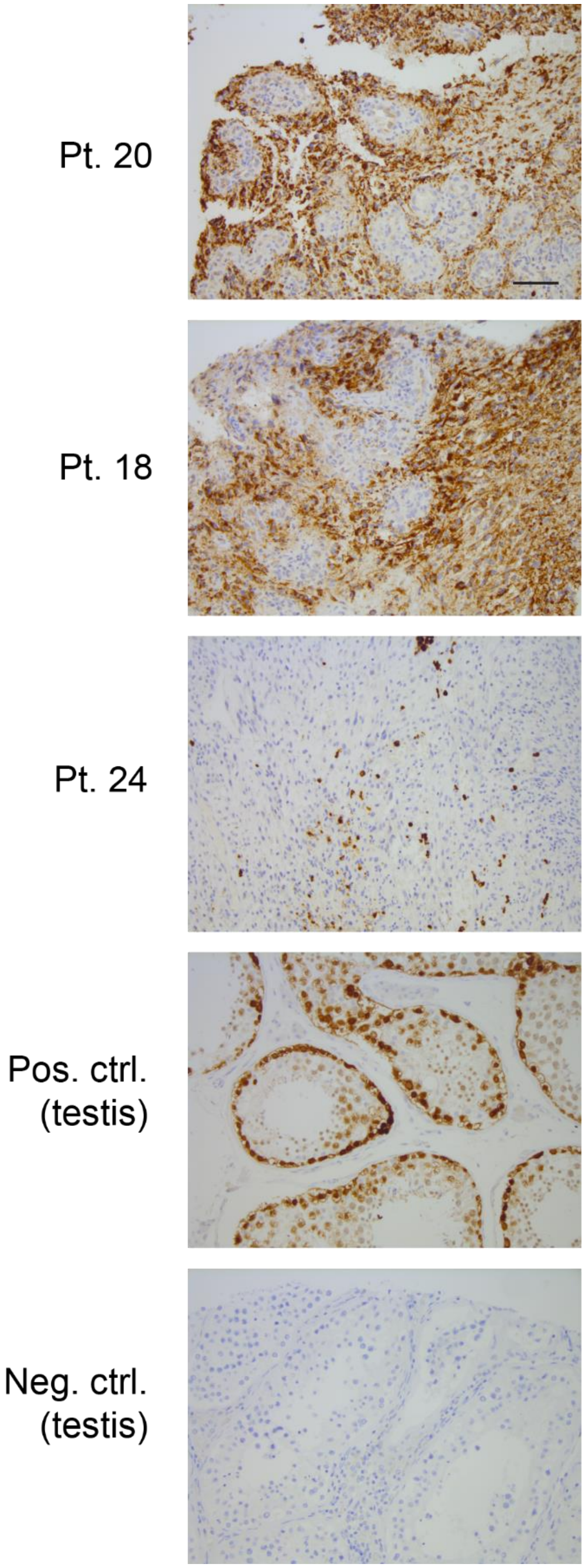

Supplementary Figure 17. Immunohistochemical analysis of MAGE-A3 in glioblastoma biopsies at diagnosis. × 200 objective; scale bar, 50 µm.

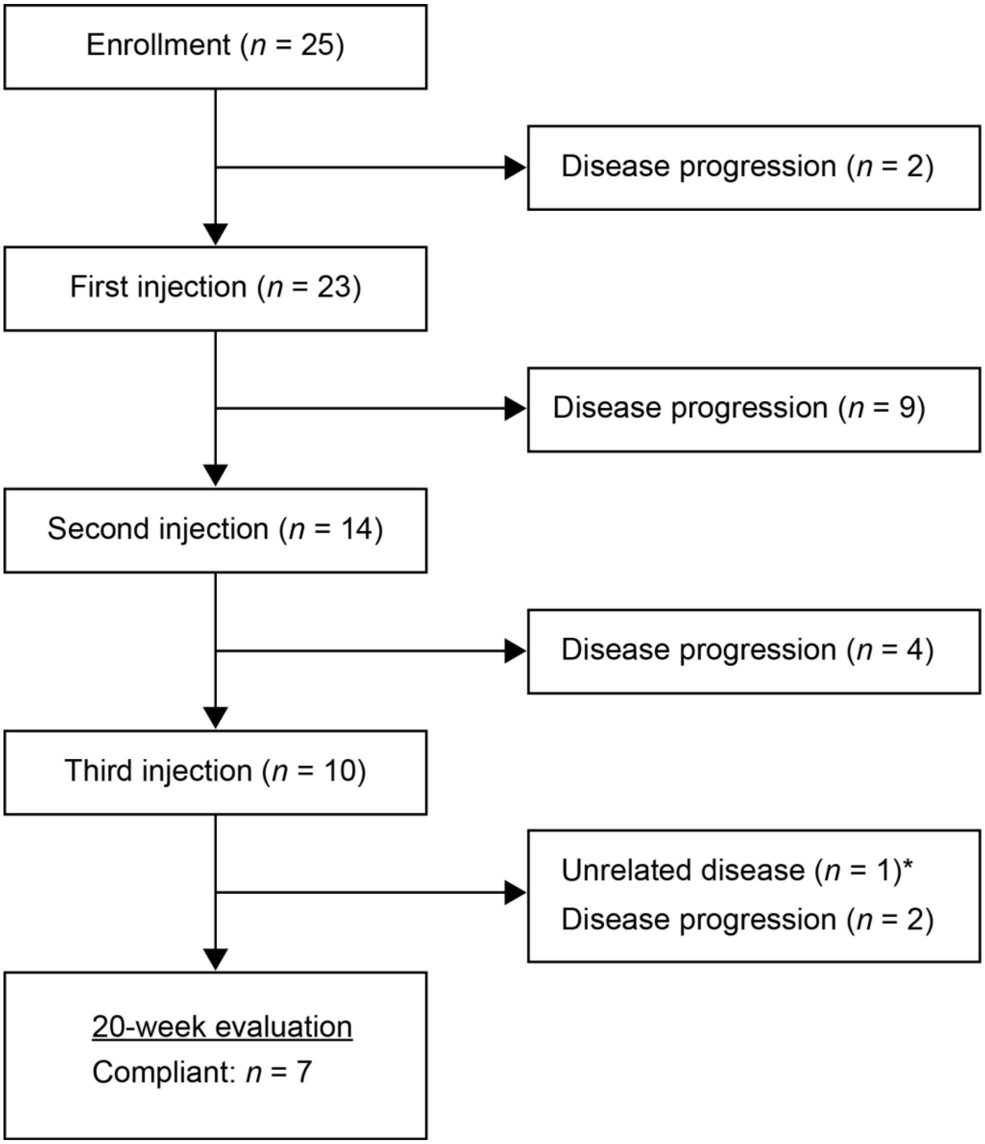

Supplementary Figure 18. Flow diagram summarizing the trial flow and grounds for exclusion from the study. Patients were removed from the study because of death or non-compliance (and subsequent death) due to disease progression. *, non-compliance due to respiratory insufficiency.

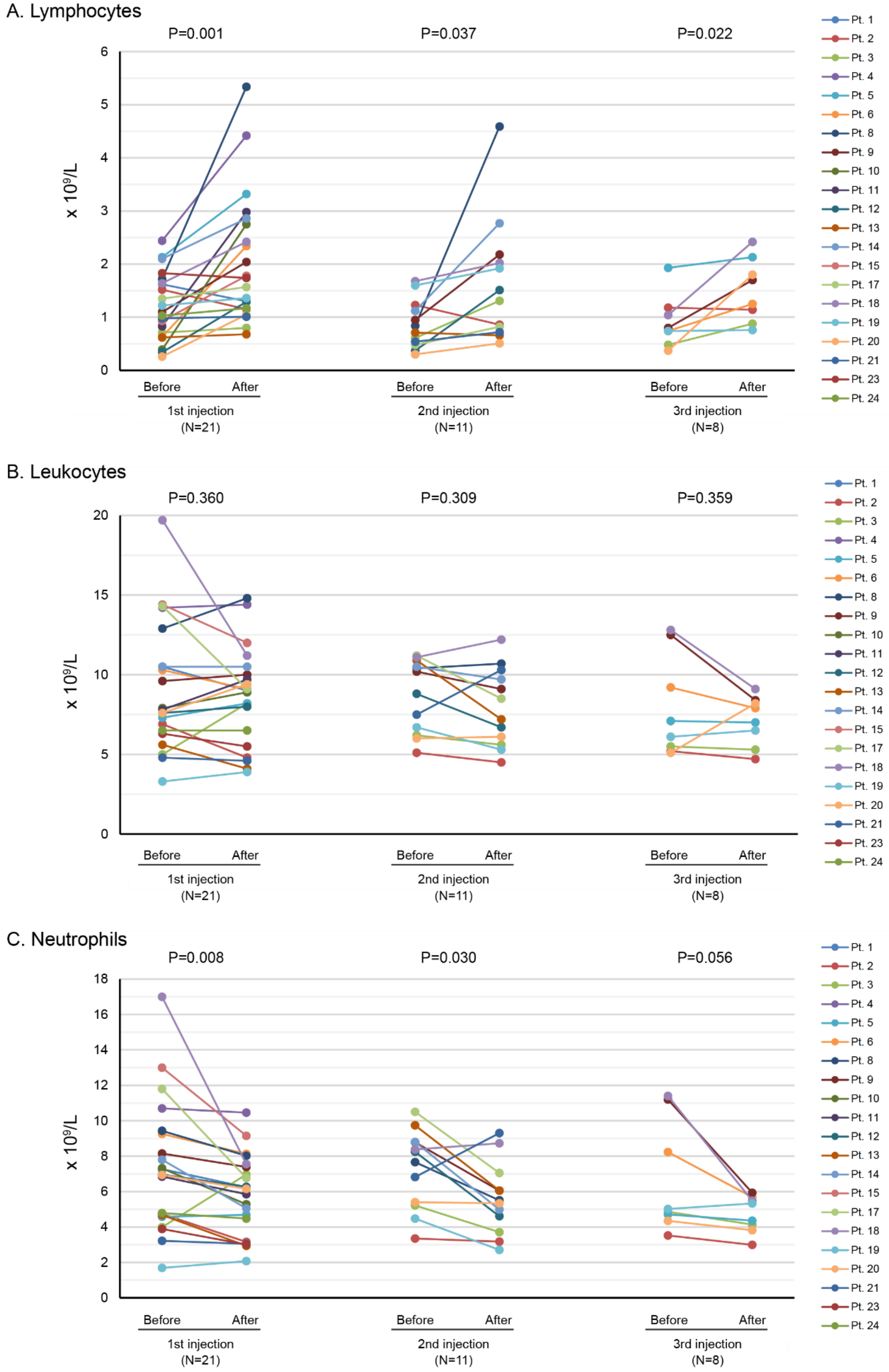
A. Lymphocytes
P=0.001
P=0.037
P=0.022
x 10⁹/L
Before
After
1st injection (N=21)
2nd injection (N=11)
3rd injection (N=8)
Pt. 1
Pt. 2
Pt. 3
Pt. 4
Pt. 5
Pt. 6
Pt. 8
Pt. 9
Pt. 10
Pt. 11
Pt. 12
Pt. 13
Pt. 14
Pt. 15
Pt. 17
Pt. 18
Pt. 19
Pt. 20
Pt. 21
Pt. 23
Pt. 24
B. Leukocytes
P=0.360
P=0.309
P=0.359
C. Neutrophils
P=0.008
P=0.030
P=0.056

Supplementary Figure 19. Transient changes in circulating lymphocyte, leukocyte and neutrophil counts after injection of cytotoxic lymphocytes.

The total numbers of leukocytes, lymphocytes and neutrophils in the patients' blood were measured immediately before and 1-2 days after injection of cytotoxic lymphocytes; shown are the highest post-injection values. The increase in lymphocytes observed after each treatment cannot be explained by the number of infused cells (median 6.8×10^7 cells per injection). The statistical comparison was performed using a paired t test.

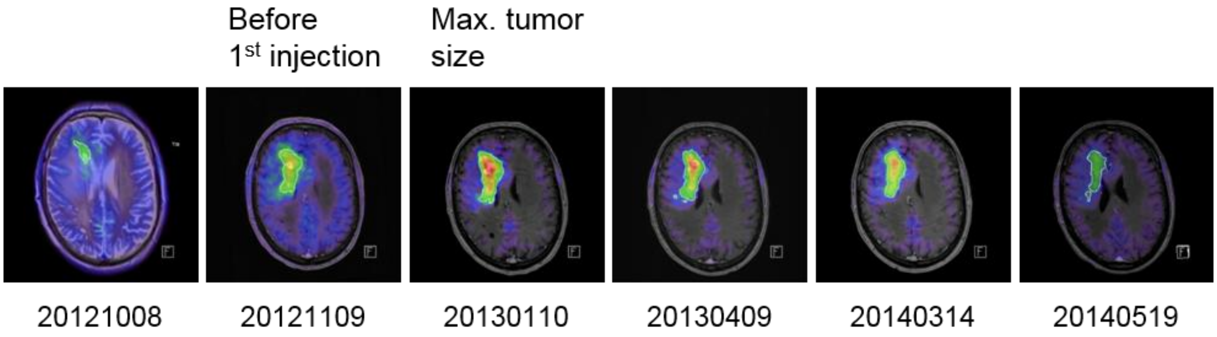

Supplementary Figure 20. Tumor response in Patient 20. Shown are FET-PET images and dates of analysis. Note the rapid tumor growth before treatment and the continued growth after initiation of treatment, reaching a maximum size 4-5 months after the first injection of autologous cytotoxic lymphocytes. The image at the far right shows reduced FET-PET volume more than one year after maximum.

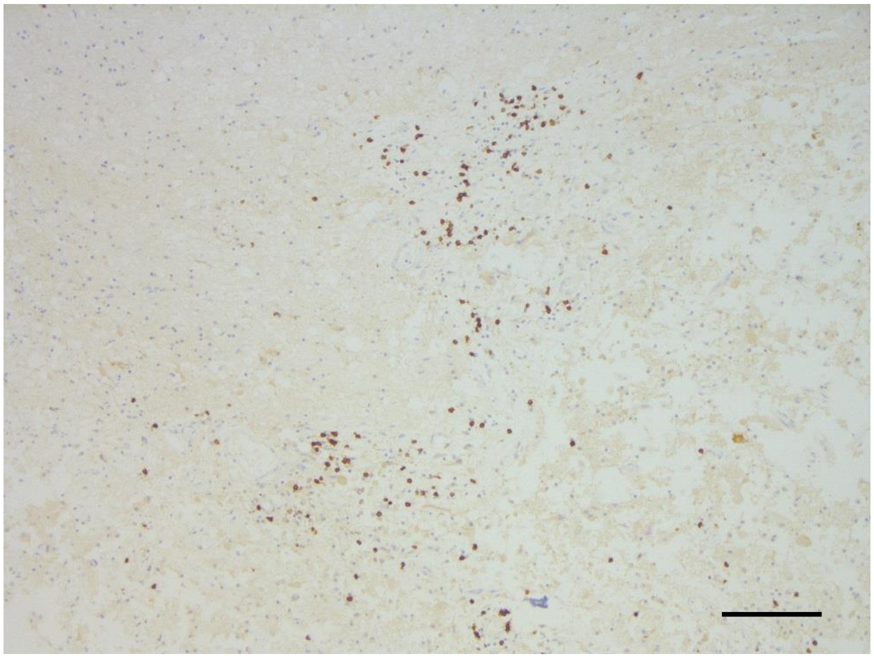

Supplementary Figure 21. T cells at the previous tumor site in Patient 6 (autopsy material). Shown is the immunohistochemical analysis of CD8 in the gliotic area surrounding the necrotic cavity (cf. Fig. 7), × 100 objective; scale bar, 100 µm.

Supplementary Table 1. Proportion of $CD4^+$ cells in non-adherent lymphocyte cultures after induction with mature DCs or PHA.

	$CD4^+$ cells (%)	
	DC*	PHA**
Exp. 1	85.1	40.1
Exp. 2	89.7	31.6
Exp. 3	81.7	18.3

* Cultured in the presence of mature DC for 7 days.
** Cultured in the presence of PHA-L (5 µg/ml) for 5 days.

Supplementary Table 2. Expression of selected surface markers on NK and T cells in the final product

Donor	CD62L		αβ-TcR		γδ-TcR		CD45R0		CD16	
	NK	T	NK	T	NK	T	NK	T	NK	T
11_17	40.8	47.8	ND	84.1	ND	15.9	ND	96.5	92.7	7.1
13_17	54	70	ND	84.1	ND	15.9	ND	90.7	84.6	9.8
16_17	87.7	86.4	ND	92.5	ND	7.5	ND	97.5	89	9.4
17_17	76.9	70.9	ND	90.5	ND	9.5	ND	ND	72.3	6.5
Average	64.8	68.8	ND	87.8	ND	12.2	ND	94.9	84.6	8.2
SD	21.3	15.9	ND	4.3	ND	4.3	ND	3.7	8.9	1.6

* Expression levels represent the percentage of the gated population. ND, not determined.

Supplementary Table 3. Stimulatory activity of $CD4^+$ and $CD8^+$ cells after treatment with 5-aza-Cdr.

	$CD4^+$	$CD8^+$
Exp. 1	270%	190%
Exp. 2	403%	190%
Exp. 3	798%	209%

Non-adherent lymphocytes (2 x 10^6) were grown in the presence of 2 x 10^6 5-aza-CdR-treated $CD4^+$ or $CD8^+$ cells for 11-12 days. Shown is the increase in cell number.

Supplementary Table 4. Lysis of MEL-ST cells expressing single CT antigens by CD56-depleted cytotoxic lymphocytes.

Donor	MAGE-A1	MAGE-A3	MAGE-A10	GAGE12	CTAG1	CTCFL
11	+	-	+	-	+	-
41	-	-	+/-	-	+	+
42	-	-	+	-	+	+
37	ND	ND	+	-	+	+
38	ND	ND	+	-	+	+

"+" indicates decrease in survival of target cells (compared to vector control) more than 25%. "+/-" indicates decrease in survival between 15% and 25%. ND – not done.

Supplementary Table 5. Baseline characteristics of glioblastoma patients (N = 25)

Pt. ID	Sex	Age	KPS	Tumor site	Primary operation	1st line treatment	2nd line Treatment	No. of reoperations (before/during immunotherapy)	Time from diagnosis to enrolment (months)	No. of lymphocyte injections*	Prednisolon dose at 1st injection (mg/day)	Survival after 1st injection (days)
1	M	61	70	T dex	Resection	RT+TMZ	BIBF 1120, BV+CPT-11	2/0	15	1	37.5	33
2	F	74	100	F dex	Resection	RT+TMZ	None	2/1	8	3 (3)	0	440
3	M	75	100	F bil	Biopsy	RT+TMZ	None	0/0	7	3 (8)	37.5	717
4	M	44	70	P dex	Resection	RT+TMZ	BV+CPT-11	0/0	10	1	37.5	39
5	M	81	90	P sin	Resection	RT	None	0/0	4	3 (1)	37.5	201
6	F	63	60	P sin	Resection	RT+TMZ	None	0/0	8	3 (5)	37.5	545
7	M	67	70	FP dex	Resection	RT+TMZ	BIBF 1120. BV+CPT-11	0/0	12	1	25	7
8	M	55	90	F dex	Resection	RT+TMZ	BV+ α-PlGF	3/1	23	2	37.5	67
9	M	71	70	T sin	Biopsy	RT+TMZ	None	0/0	5	3	37.5	115
10	M	71	80	F dex	Resection	RT+TMZ	BV+CPT-11	1/0	10	1	37.5	86
11	M	64	100	F dex	Resection	RT+TMZ	BV	1/1	10	1	0	72
12	M	60	70	T dex	Resection	RT+TMZ	BV+ α-PlGF	0/0	12	2	25	63
13	F	59	100	FT dex	Resection	RT+TMZ	BV+CPT-11	0/0	24	2	25	105
14	F	79	60	F dex	Resection	RT+TMZ	None	0/0	15	3	25	144
15	M	56	90	TP sin	Resection	RT+TMZ	BV+CPT-11	0/0	15	1	37.5	28
16	M	62	70	T sin	Resection	RT+TMZ	None	0/0	10	0	NA	NA
17	M	64	60	FT sin	Resection	RT+TMZ	BV+CPT-11	2/0	12	1	37.5	36
18	M	64	100	T sin	Resection	RT+TMZ	BV+CPT-11	0/1	36	3 (2)	10	267
19	M	39	100	F dex	Resection	RT+TMZ	None	3/0	16	3	0	122
20	M	56	100	F dex	Resection	RT+TMZ	BV+CPT-11	0/1	14	3 (18)	37.5	1384
21	F	47	100	T dex	Resection	RT+TMZ	BV+CPT-11	0/0	31	2	37.5	56
22	M	47	70	F sin	Resection	RT+TMZ	BV+CPT-11	1/1	31	1	37.5	19
23	F	66	60	T sin	Resection	RT+TMZ	BV+CPT-11	0/0	23	1	25	43
24	M	65	90	P dex	Resection	RT+TMZ	BV+CPT-11	2/1	13	3 (1)	0	164
25	M	42	60	FP sin	Resection	RT+TMZ	BV+CPT-11. BV+ α-PlGF	3/0	36	0	NA	NA

*) Number in parenthesis indicates additional injections after 20-week trial. BV = bevacizumab; CPT-11 = irinotecan; TMZ = temozolomide; RT = radiation therapy; α-PlGF = anti placental growth factor antibody; KPS = Karnofsky Performance Score; F = frontal; T = temporal; P = parietal; sin = sinister; dex = dexter; bil = bilateral; NA, not applicable

Supplementary Table 6. Expression of CTCFL and MAGE-A3 in diagnostic tumor biopsies (immunohistochemical analysis)

Patient ID	CTCFL	MAGE-A3
2	+	++
3	++	++++
5	+	+++
6	0	+++
7	0	++
8	+	++++
9	0	+++
10	+	++++
11	0	0
12	+++	++
16	+++	++
17	+++	++++
18	+	+++
19	0	0
20	+++	++++
21	+	+++
22	0	+++
23	+	+++
24	++	++
25	0	0

Supplementary Table 7. Induction of MAGE-antigen mRNA expression by 5-aza-CdR in T_H cells from patients with glioblastoma

Patient ID*	MAGE-A1	MAGE-A3	MAGE-A4	MAGE-A6	MAGE-A10	MAGE-A12
2 (a)	+	+	+	+	+	+
2 (b)	+	+	+	+	+	+
2 (c)	-	(+)	(+)	-	-	(+)
2 (d)	+	+	+	(+)	(+)	+
2 (e)	+	+	+	+	+	+
2 (f)	+	+	+	-	-	+
3 (b)	+	+	+	+	(+)	+
3 (c)	+	+	+	+	+	+
3 (d)	+	+	+	+	(+)	+
3 (e)	+	+	+	+	+	+
3 (f)	+	+	+	+	+	+
3 (g)	+	+	+	+	+	+
3 (h)	+	+	+	+	+	+
4 (a)	+	+	+	(+)	(+)	+
4 (b)	+	+	+	+	+	+
5 (a)	+	(+)	+	-	-	+
5 (b)	+	+	+	+	+	+
5 (d)	+	+	+	+	+	+
6 (a)	+	+	+	+	(+)	+
6 (b)	+	+	+	+	+	+
6 (c)	+	+	+	+	+	+
6 (d)	+	+	+	+	+	+
6 (f)	+	+	+	+	+	+
6 (g)	+	+	+	+	+	+
7 (a)	+	+	+	+	+	+
8 (a)	+	+	+	+	+	+
8 (b)	+	+	+	+	+	+
9 (a)	+	+	+	(+)	-	+
9 (c)	+	+	+	+	+	+
9 (d)	+	+	+	+	+	+
11 (a)	(+)	(+)	+	-	-	+
11 (b)	+	+	+	+	+	+
12 (a)	+	+	+	+	+	+
12 (c)	+	+	+	+	+	+
13 (a)	+	+	+	+	+	+
13 (b)	+	+	+	+	+	+
14 (a)	+	+	+	+	+	+
14 (b)	+	+	+	+	+	+
14 (c)	+	+	+	+	+	+
15 (a)	+	+	+	+	+	+
16 (a)	+	+	+	+	+	+
18 (a)	+	+	+	(+)	-	+
18 (c)	+	-	+	-	-	+

18 (d)	+	+	+	+	+	+
18 (e)	+	+	+	+	+	+
18 (f)	+	+	+	+	+	+
19 (a)	+	+	+	+	+	+
19 (b)	+	+	+	(+)	+	+
19 (c)	+	+	+	-	(+)	+
19 (d)	+	+	+	+	+	+
20 (a)	+	+	+	+	(+)	+
20 (b)	+	+	+	-	-	+
20 (c)	+	+	+	+	(+)	+
20 (d)	+	+	+	+	+	+
20 (f)	+	+	+	+	+	+
20 (j)	+	+	+	+	+	+
20 (k)	+	+	+	+	+	+
21 (b)	+	+	+	(+)	-	+
22 (a)	+	+	+	+	+	+
23 (a)	+	-	+	-	-	+
23 (b)	+	+	+	+	+	+
24 (a)	+	+	+	+	+	+
24 (b)	+	+	+	+	+	+
24 (c)	+	+	+	+	+	+
24 (d)	+	+	+	+	+	+
25 (a)	+	+	+	+	+	+

+, indicates a visible band in EtBr-stained agarose gels after 35 cycles of amplification. (+), indicates weak or inconsistent signals. *, letters in parentheses indicate preparations from repeat blood samples from the same patient during the course of the trial.

Supplementary Table 8. Cell therapy products administered in the phase 1 glioblastoma trial.

Patient ID*	No. of cells	Viability (%)	% T cells
1a	176,000,000	93	73
2a	10,000,000	94	60
2b	22,000,000	95	51
2c	76,000,000	96	55
3a	100,000,000	98	83
3b	51,000,000	95	79
3c	52,500,000	92	84
4a	76,000,000	95	79
5a	5,400,000	96	66
5b	46,000,000	95	93
5c	53,000,000	92	90
6a	18,000,000	90	70
6b	10,000,000	95	66
6c	10,000,000	91	82
7a	98,500,000	95	72
8a	26,000,000	98	96
8b	20,000,000	98	79
9a	20,000,000	98	91
9b	523,000,000	93	62
9c	150,000,000	93	91
10a	27,000,000	96	68
11a	134,000,000	94	22
12a	9,400,000	92	80
12b	11,000,000	96	75
13a	21,000,000	95	40
13b	45,000,000	97	46
14a	471,000,000	86	8
14b	96,000,000	88	16
14c	170,000,000	97	22
15a	68,000,000	83	27
17a	8,400,000	100	51
18a	53,000,000	90	38
18b	300,000,000	98	17
18c	281,000,000	96	33
19a	250,000,000	96	67
19b	147,000,000	95	71
19c	597,000,000	94	77
20a	16,800,000	94	70
20b	89,000,000	97	73
20c	62,000,000	97	61
21a	440,000,000	99	91
21b	221,000,000	94	91
22a	1,857,000,000	97	20
23a	179,000,000	88	63
24a	114,000,000	100	44
24b	77,000,000	91	36
24c	48,000,000	93	47

* Letters indicate preparations from repeat blood samples from the same patient during the course of the trial.

Supplementary Table 9. Adverse events reported during the 20-week study period after first injection of therapeutic cells

**Adverse event** (preferred terms)	**Severity**	**Number of events**	**Patients at risk (23 treated patients)** n (%)
Nausea	Moderate	4	3 (13%)
Dizziness	Moderate	3	3 (13%)
Dyspnea	Moderate	2	2 (9%)
Pneumonia	Moderate	2	2 (9%)
Tiredness	Moderate	2	2 (9%)
Epilepsy	Moderate	2	2 (9%)
Aphasia	Mild	2	2 (9%)
Unconsciousness	Severe	1	1 (4%)
Drop in blood pressure	Moderate	1	1 (4%)
Back pain	Moderate	1	1 (4%)
Shoulder pain	Moderate	1	1 (4%)
Impaired Vision	Moderate	1	1 (4%)
Increased nausea	Moderate	1	1 (4%)
Appetite loss	Moderate	1	1 (4%)
Increased tiredness	Moderate	1	1 (4%)
Pale	Moderate	1	1 (4%)
Sinus tachycardia	Moderate	1	1 (4%)
Thrombosis leg	Moderate	1	1 (4%)
Confusional state	Mild	1	1 (4%)
Finger cramps	Mild	1	1 (4%)
Leg cramp	Mild	1	1 (4%)
Leg pain	Mild	1	1 (4%)

Supplementary Table 10. Serious adverse events reported during the 20-week study period after first injection of therapeutic cells

SAE Term	SAEs total	CTCAE-grading				
		1	2	3	4	5
Disease progression GBM	9		2	3		4
Disease progression NOS	2					2
General physical health deterioration	2				1	1
Neurological status deterioration	1		1			
Diarrhoea NOS	1		1			
Seizures (incl. subtypes)	1		1			
Epileptic aura	1		1			
Hydrocephalus	1		1			
Hemiparesis	1		1			
Paresis	1		1			
Cerebral vascular lesion (NOS)	1				1	
Pneumonia	1				1	
Respiratory insufficiency	1					1

NOS, not otherwise specified

Supplementary Table 11. Additional injections of cytotoxic lymphocytes under approved compassionate use

No. Injection*	Patient ID						
	2	3	5	6	18	20	24
4	154	154	154	168	175	140	105
5	189	203		238	203	175	
6	231	238		448		217	
7		469		483		245	
8		525		532		287	
9		553				350	
10		644				392	
11		721				434	
12						525	
13						574	
14						707	
15						763	
16						933	
17						1064	
18						1162	
19						1232	
20						1314	
21						1365	

Numbers indicate days after 1st injection of cytotoxic lymphocytes.
*) Additional injections were offered until death or non-compliance due to GBM progression (Pts. 2, 3, 18, 20 and 24), progression of other malignancy (Pt. 5), or death caused by an unrelated heart condition (Pt. 6).

Supplementary Table 12. CT-antigen expression in cancer cell lines used in this study

Cell line	MAGE-A1	MAGE-A3	MAGE-A4	MAGE-A6	MAGE-A10	MAGE-A12	GAGE-3-7	CTCFL	CTAG1
MDA-MB-231	+	+	+	+	-	+	+	+	-
MCF-7	+	-	+	-	-	+	+	+	-
CAMA-1	-	+	-	+	-	+	+	-	+
FM3.29	+	+	+	+	+	+	+	+	+
T47D	+	+	-	+	+	+	+	-	+
U-87	-	+	+	-	+	+	+	+	-
MEL-ST	-	-	-	-	-	-	-	-	-

+, indicates a visible band in EtBr-stained agarose gels after 35 (MAGE), 37 (GAGE 3-7 and CTAG1), and 40 (CTCFL) cycles of amplification.

Supplementary Table 13. Antibodies

Target	Conjugation	Catalog No.	Supplier	Analysis	Dilution
CTCFL	-	11074-2-AP	ProteinTech	IHC	1:150
MAGEA3	-	60054-1-Ig	ProteinTech	IHC	1:200
Ki-67	-	M7240	Dako	IHC	1:200
p53	-	790-2912	Ventana/Roche	IHC	1:200
Nestin	-	MAB5326	Millipore	IHC	1:800
Olig2	-	387M	Cell Marque	IHC	1:100
MAP2	-	M9942	Sigma	IHC	1:12800
CD68	-	M0876	Dako	IHC	1:100
CD8	-	790-4460	Ventana/Roche	IHC	Undiluted
HLA-ABC	-	CLHLA-01NA	Cedarlane	Blocking	
IgG2a	-	16-4724-85	eBioscience	Blocking	
MAGE	-	Clone 57B*	G.C. Spagnoli	IB	1:15
				CbELISA	1:10
				ELISA	1:20
IgG_1, κ	-	554121	BD Biosciences	CbELISA	1:200
				ELISA	1:400
MAGEA3	-	ABC468	Merck	ELISA	1:800
CD3	FITC	IM1281	Beckman	FCM	As recommended
CD4	FITC	555346	BD Biosciences	FCM	As recommended
CD8	PE	555635	BD Biosciences	FCM	As recommended
CD16	PE-Cy5	555408	BD Biosciences	FCM	As recommended
CD27	PE	555441	BD Biosciences	FCM	As recommended
CD40	FITC	555588	BD Biosciences	FCM	As recommended
CD45RO	PE	561889	BD Biosciences	FCM	As recommended
CD56	PE	555516	BD Biosciences	FCM	As recommended
CD62L	PE-Cy5	IM2655	Beckman	FCM	As recommended
CD70	FITC	555834	BD Biosciences	FCM	As recommended
CD80	FITC	557226	BD Biosciences	FCM	As recommended
CD83	PE	556855	BD Biosciences	FCM	As recommended
CD86	PE	555658	BD Biosciences	FCM	As recommended
HLA-DR, DP, DQ	FITC	555558	BD Biosciences	FCM	As recommended
MIC A/B	PE	558352	BD Biosciences	FCM	As recommended
αβ-TcR	PE	B49177	Beckman	FCM	As recommended
γδ-TcR	PE	555717	BD Biosciences	FCM	As recommended

*, 25 μg/ml. IHC: immunohistochemistry; IB, immunoblotting; CbELISA, cell-based ELISA; FCM, flow cytometry; FITC, fluorescein isothiocyanate; PE, phycoerythrin.

Supplementary Table 14. Primer sequences for RT-PCR

Gene	Primer sequences (5'-3')	Length (bp)	Reference
MAGE-A1	GATTCCCTGGAGGCCACAG<br>CCTCACTGGGTTGCCTCTGTC	341	2
MAGE-A3	ACCAGAGGCCCCCGGAGGAG<br>TCCGACGACACTCCCCAGCAT	534	2
MAGE-A4	GAGCAGACAGGCCAACCG<br>AAGGACTCTGCGTCAGGC	446	3
MAGE-A6	AGGACCAGAGGCCCCC<br>GGATGATTATCAGGAAGCCTGT	576	
MAGE-A10	CACAGAGCAGCACTGAAGGAG<br>CTGGGTAAAGACTCACTGTCTGG	485	3
MAGE-A12	TGGAAGTGGTCCGCATCG<br>GCCCTCCACTGATCTTTAGCAA	390	3
CTAG1	CCCACCGCTTCCCGTG<br>GGCCACTCGTGCTGGGA	272	4
GAGE3-7	GACCAAGGCGCTATGTAC<br>CCATCAGGACCATCTTCA	244	5
CTCFL	GAAAAGGCCAAATCTACAAAAAATC<br>GACCCTTTGTGGCTTCCTTCAG	1074	6
GADPH	AGGGGGGAGCCAAAAGGG<br>GAGGAGTGGGTGTCGCTGTTG	540	2

Supplementary Table 15. Primer sequences for bisulfite pyrosequencing

Gene	Primer sequences (5'-3')		Reference
CTAG1	Fw	GGGGTTTTTTAGGGTAGGAAGTA	7
	Rev	[Btn]-CCCTAAACCATCAAAAATACCA	
	Seq	AGTAGGAGTTTTGAGGAT	
MAGEA1	Fw	TAATTTTGATGTTTATTYGTTTAGTTAT	7
	Rev	[Btn]-CAAAACCTAAATCAAATTCCTT	
	Seq	TTTTTATTTAGGTAGGATT	
MAGEA3/6	Fw	GGTAGAATTTAGTTTTATTTTTG	
	Rev	[Btn]-ACTACAAAACTTACCTCCTCAC	
	Seq	TATTTTTGTTYGGAATTTAGGGTA	

Supplementary Table 16. Primer sequences for MS-MCA

Gene	Primer sequences (5'-3')
MAGEA3/6	GGTAGAATTTAGTTTTATTTTTGT<br>CTCAAAACCTTACCTCCTCA

Supplementary References

1. Pauleit,D. *et al.* O-(2-[18F]fluoroethyl)-L-tyrosine PET combined with MRI improves the diagnostic assessment of cerebral gliomas. *Brain* **128**, 678-687 (2005).

2. thor Straten,P., Kirkin,A.F., Seremet,T., & Zeuthen,J. Expression of transporter associated with antigen processing 1 and 2 (TAP1/2) in malignant melanoma cell lines. *Int. J. Cancer* **70**, 582-586 (1997).

3. De Plaen,E. *et al.* Structure, chromosomal localization, and expression of 12 genes of the MAGE family. *Immunogenetics* **40**, 360-369 (1994).

4. Lethe,B. *et al.* LAGE-1, a new gene with tumor specificity. *Int. J. Cancer* **76**, 903-908 (1998).

5. De Backer,O. *et al.* Characterization of the GAGE genes that are expressed in various human cancers and in normal testis. *Cancer Res.* **59**, 3157-3165 (1999).

6. Vatolin,S. *et al.* Conditional expression of the CTCF-paralogous transcriptional factor BORIS in normal cells results in demethylation and derepression of MAGE-A1 and reactivation of other cancer-testis genes. *Cancer Res.* **65**, 7751-7762 (2005).

7. Rao,M. *et al.* Inhibition of histone lysine methylation enhances cancer-testis antigen expression in lung cancer cells: implications for adoptive immunotherapy of cancer. *Cancer Res.* **71**, 4192-4204 (2011).
